# Detecting Selection on Temporal and Spatial Scales: A Genomic Time-Series Assessment of Selective Responses to Devil Facial Tumor Disease

**DOI:** 10.1371/journal.pone.0147875

**Published:** 2016-03-01

**Authors:** Anna Brüniche-Olsen, Jeremy J. Austin, Menna E. Jones, Barbara R. Holland, Christopher P. Burridge

**Affiliations:** 1 School of Biological Sciences, University of Tasmania, Private Bag 55, Hobart 7001, Tasmania, Australia; 2 School of Earth & Environmental Sciences, University of Adelaide, North Terrace Campus, South Australia, 5005, Australia; 3 School of Physical Sciences, University of Tasmania, Private Bag 37, Hobart, Tasmania, 7001, Australia; Aristotle University of Thessaloniki, GREECE

## Abstract

Detecting loci under selection is an important task in evolutionary biology. In conservation genetics detecting selection is key to investigating adaptation to the spread of infectious disease. Loci under selection can be detected on a spatial scale, accounting for differences in demographic history among populations, or on a temporal scale, tracing changes in allele frequencies over time. Here we use these two approaches to investigate selective responses to the spread of an infectious cancer—devil facial tumor disease (DFTD)—that since 1996 has ravaged the Tasmanian devil (*Sarcophilus harrisii*). Using time-series ‘restriction site associated DNA’ (RAD) markers from populations pre- and post DFTD arrival, and DFTD free populations, we infer loci under selection due to DFTD and investigate signatures of selection that are incongruent among methods, populations, and times. The lack of congruence among populations influenced by DFTD with respect to inferred loci under selection, and the direction of that selection, fail to implicate a consistent selective role for DFTD. Instead genetic drift is more likely driving the observed allele frequency changes over time. Our study illustrates the importance of applying methods with different performance optima e.g. accounting for population structure and background selection, and assessing congruence of the results.

## Introduction

Identification of genetic variants that are advantageous to a population is of immense interest in evolutionary biology, but disentangling whether a change in allele frequency of a genetic variant is driven by selection or genetic drift poses a problem. Traditionally, allele frequency approaches to the inference of loci under selection have employed a ‘single time point’ perspective based on measures of population differentiation (e.g. *F*_ST_, site frequency spectrum (SFS), linkage disequilibrium (LD)) [1–3]. These approaches suffer from bias when applied to non-equilibrium situations (e.g. population structure) resulting in large numbers of ‘false positives’ or ‘false negatives’ [4]. Extensions to ‘single time point’ approaches—accounting for differences in demographic history—are available [2, 5]. Most rely on a proportion of the loci being neutral in order to detect loci under selection, an assumption that is not always met. Novel ‘multiple time points’ methods have been developed that rely on sampling a population through time—investigating temporal changes in allele frequencies. These approaches have an advantage in that trajectories of allele frequencies are traced, providing valuable information on the underlying forces responsible for their fate [6–9]. The ‘multiple time points’ approaches enables genetic drift and selection to be disentangled [8], while increasing power for the inference of population genetic parameters [4].

Infectious diseases are arguably a major selective force shaping species’ genetic diversity [10]. These diseases are recognized as a serious threat to wildlife [11], and identifying loci potentially associated with disease resistance or longevity with disease are of great interest in conservation biology [12]. One species that has been severely affected by infectious disease is the largest marsupial carnivore, the Tasmanian devil (*Sarcophilus harrisii*). Since its emergence in 1996, devil facial tumor disease (DFTD) has spread from Mt. William National Park in northeast Tasmania to the majority of the species’ island range, causing 90% decline in devil abundance [13]. DFTD manifests as large tumors, primarily around the face and mouth, and is almost 100% fatal within six months of infection. DFTD is unique in that live cells are the infectious agent. The cancer cells are not passed from parents to offspring, but transferred via injurious contact between individuals, primarily during the mating season [14]. The transferred DFTD cells avoid recognition by the immune system as antigens are not represented on the cell surface [15]. The tumor cells thus evade an immune response in infected devils and the cells are able to develop into tumors. Strong frequency-dependent transmission of DFTD represents a serious extinction threat for the species [16].

Tasmanian devil populations have responded to DFTD directly via decrease in population sizes, and possibly directly or indirectly with respect to changes in behavior and life history [17]. Female devils in DFTD affected populations have reduced dispersal distance [18], while immigration from non-DFTD populations to DFTD populations has increased [19]. In devil populations with long-term DFTD presence, greater percentages of one-year old devils breed [17], a response known in other disease-affected species [20]. With the reduction in the number of lifetime breeding events—from iteroparity towards semelparity—precocial breeding may buffer the species against extinction, although this reproductive compensation has not been sufficient to halt population decline [21]. Selection under DFTD may be very strong due to the cancer’s almost 100% mortality within six months of infection, such that precocial breeding, in particular, would be strongly favored [17]. However, the small effective population size of devils (*N*_e_<500) [19, 22] makes them prone to genetic drift, and advantageous alleles under DFTD might be lost and detrimental alleles fixed by chance [23]. Conservation of species affected by infectious disease such as DFTD relies on correct identification of alleles associated with increased fitness, thus robust results are needed when implementing conservation plans based on genomic approaches [24, 25].

In this study we use ‘single time point’ and ‘multiple time points’ approaches to investigate selective responses of devils to DFTD at the genomic level. The novelty of this study is that we have detailed information of the Tasmanian devil’s demographic history, together with temporal sampling from the species’ geographic range and timing information on the spread of DFTD. The ideal way to identify loci under selection—in response to disease—is to have samples from before and after the disease enters a population, and to sample multiple populations along with replicates and non-affected populations, the latter to act as controls against other environmental changes that may be influential. We accomplish this by investigating thousands of Restriction-site associated DNA (RAD) markers from six Tasmanian devil populations sampled from 1999 to 2013, spanning both pre- and post DTFD arrival, as well as non-infected control populations. Our sampling enables more rigorous assessments of selection, in that we can test for (i) an increase in the number of outlier loci with increasing duration of exposure to disease at a population, (ii) congruence in the outlier loci identified among multiple DFTD-affected populations, and (iii) congruence between the loci identified as being under selection with ‘single time point’ (BAYESCAN v2.1 [5]) and ‘multiple time points’ (WFABC v1.0 [8, 9]) approaches.

## Results

### RAD processing and genetic diversity

**Fig 1:**
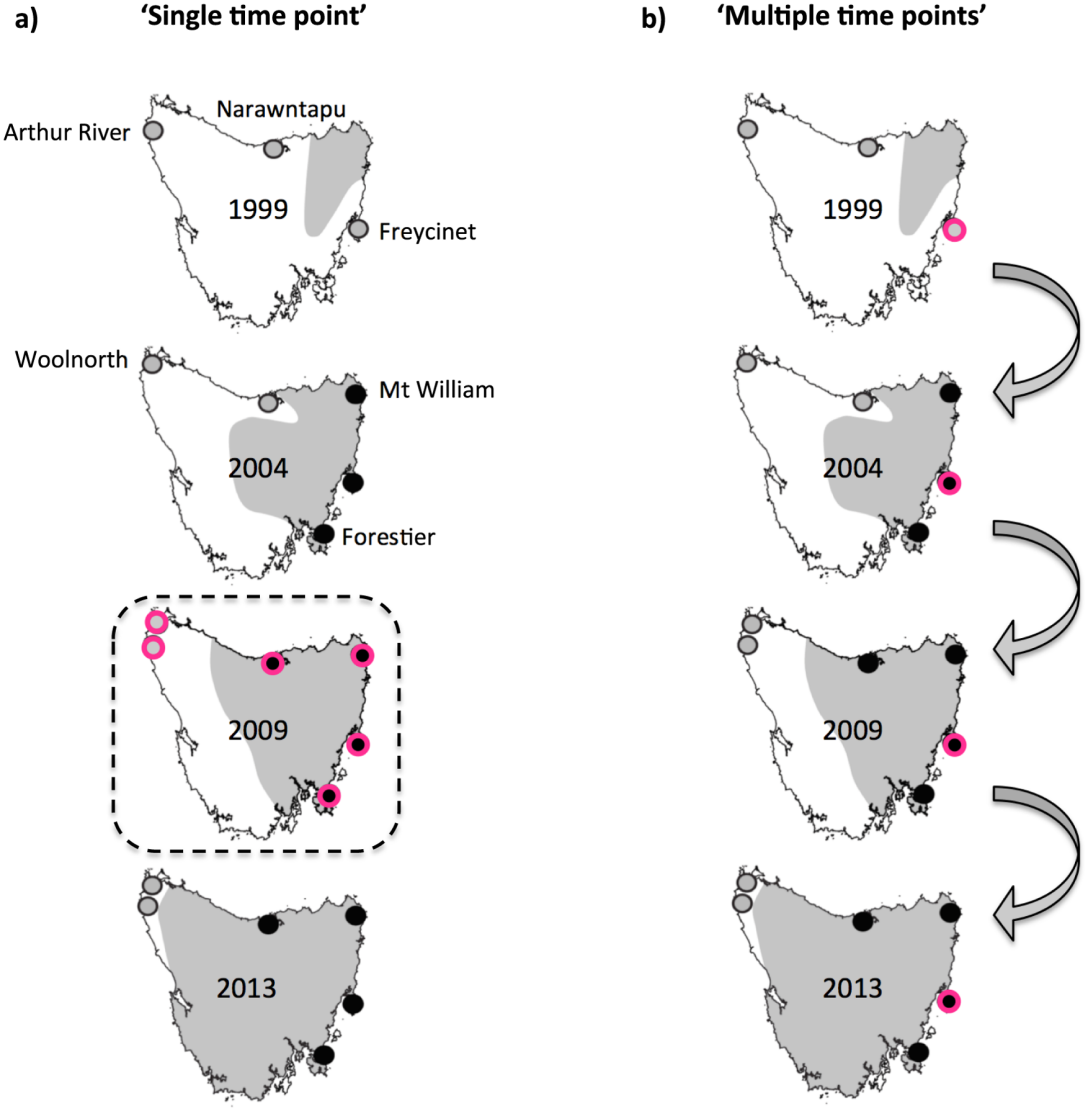
Selection approaches and sampling locations 1999, 2004, 2009 and 2013. Dots indicate locations sampled within the year as indicated on the corresponding map. Single examples of each approach are illustrated by pink dots for (A) ‘Single time point’ analyses represented by all populations sampled in a given year, with a single example represented by the dashed line, and (B) ‘Multiple time points’ approach where one population is sampled at multiple time points represented by arrows. Presence or absence of DFTD at the time of sampling is indicated by black and grey dots, respectively. The area affected by DFTD is indicated with shaded area.

DNA samples from 527 Tasmanian devils collected between 1999 and 2013 were analyzed. The samples were from six populations; one that was affected, three of which became infected with DFTD after initial samples were taken and two non-infected control populations (Fig 1). We sequenced 3×10^3^ RAD tags of 100 bp at 25x coverage. These aligned to chromosomes 1–6, the X chromosome, and super-contigs without specific genome location [26]. Out of the 2281 SNPs identified, 1482 were left after removing those representing indels or SNPs located on the X chromosome (due to lack of sex information on all individuals). A single SNP was retained from RAD markers with multiple SNPs. The data are available from the Dryad Digital Depository (doi:10.5061/dryad.86bq5). Genetic diversity was evenly distributed across the six Tasmanian devil populations (S1 File). Mean number of alleles per locus per population (*A*) was 1.88–1.98, with some loci being fixed in individual populations, observed heterozygosity (*H*_O_) was 0.393–0.455, and expected heterozygosity (*H*_E_) was 0.315–0.356. The number of private alleles was low (*A*_P_ = 1–11) and only four populations had private alleles. We found no evidence of change in genetic diversity over time in relation to DFTD (Fig 1; S1 File). Over time Woolnorth increased in *A* and Arthur River decreased in *H*_O_ (S1 File).

### Selection–‘single time point’

**Fig 2:**
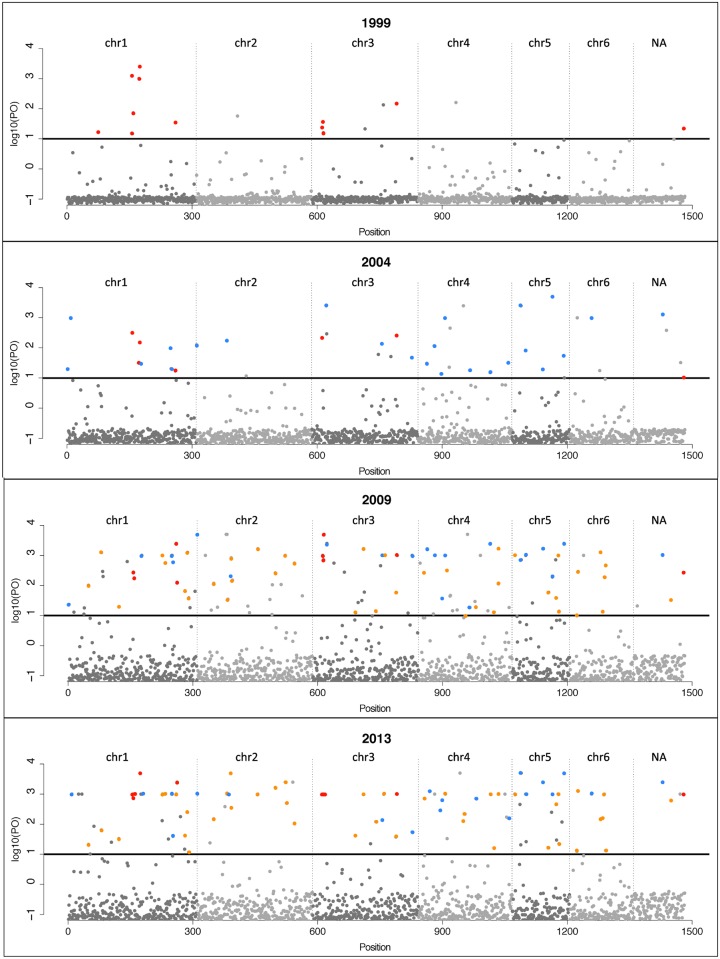
Support for SNPs under selection in Tasmanian devil populations. Results from BAYESCAN based on prior odds (PO = 10). Black vertical lines divide the SNPs into chromosome assignment. For all SNPs Bayesian *P*-values on a log_10_ scale are given. Black horizontal lines correspond to a threshold of *P* = 0.01 for positive selection. Loci under selection in a minimum of one consecutive year are shown with colored dots that indicate the first time period in which they were identified as being under selection.

All populations contained loci that appear to be under positive selection based on a ‘single time point’ approach implemented in BAYESCAN (Fig 2). These were identified for all chromosomes (Table 1a; Fig 2). No loci were identified as being under negative selection using this method (Table 1a). In total 274 SNPs were identified as being under selection (S2 File), and the number of SNPs under selection increased over time (S2 File).

**Table 1:** Number of SNPs under selection for a) each year as detected with BAYESCAN, and the individual population as detected with WFABC for b) a small (*N*_e_ = 50), and c) and a large (*N*_e_ = 500) effective population size. For each method the number of SNPs under positive and negative selection are given along with their distribution among chromosomes (chr1–chr6) where identifiable, or otherwise listed without chromosome location (NA).

	Location/ year	Positive selection	Negative selection	Chr1	Chr2	Chr3	Chr4	Chr5	Chr6	NA
**a)**	1999	17	-	7	1	6	1	-	-	2
	2004	43	-	9	3	8	10	6	3	4
	2009	113	-	24	22	20	24	12	7	4
	2013	101	-	30	14	12	18	16	7	4
**b)**	Woolnorth	2	-	-	-	2	-	-	-	-
	Arthur River	1	1	-	1	1	-	-	-	-
	Narawntapu	-	1	-	1	-	-	-	-	-
	Mt William	3	-	-	2	-	-	1	-	-
	Freycinet	5	1	2	-	3	1	1	-	-
	Forestier	3	1	-	2	-	-	-	2	-
**c)**	Woolnorth	39	7	11	5	11	10	3	5	1
	Arthur River	27	32	9	14	18	9	4	2	3
	Narawntapu	33	21	14	17	6	5	3	5	4
	Mt William	62	34	23	21	9	18	13	8	4
	Freycinet	37	23	14	12	18	3	6	5	2
	Forestier	37	43	26	16	10	11	4	9	4

**Fig 3:**
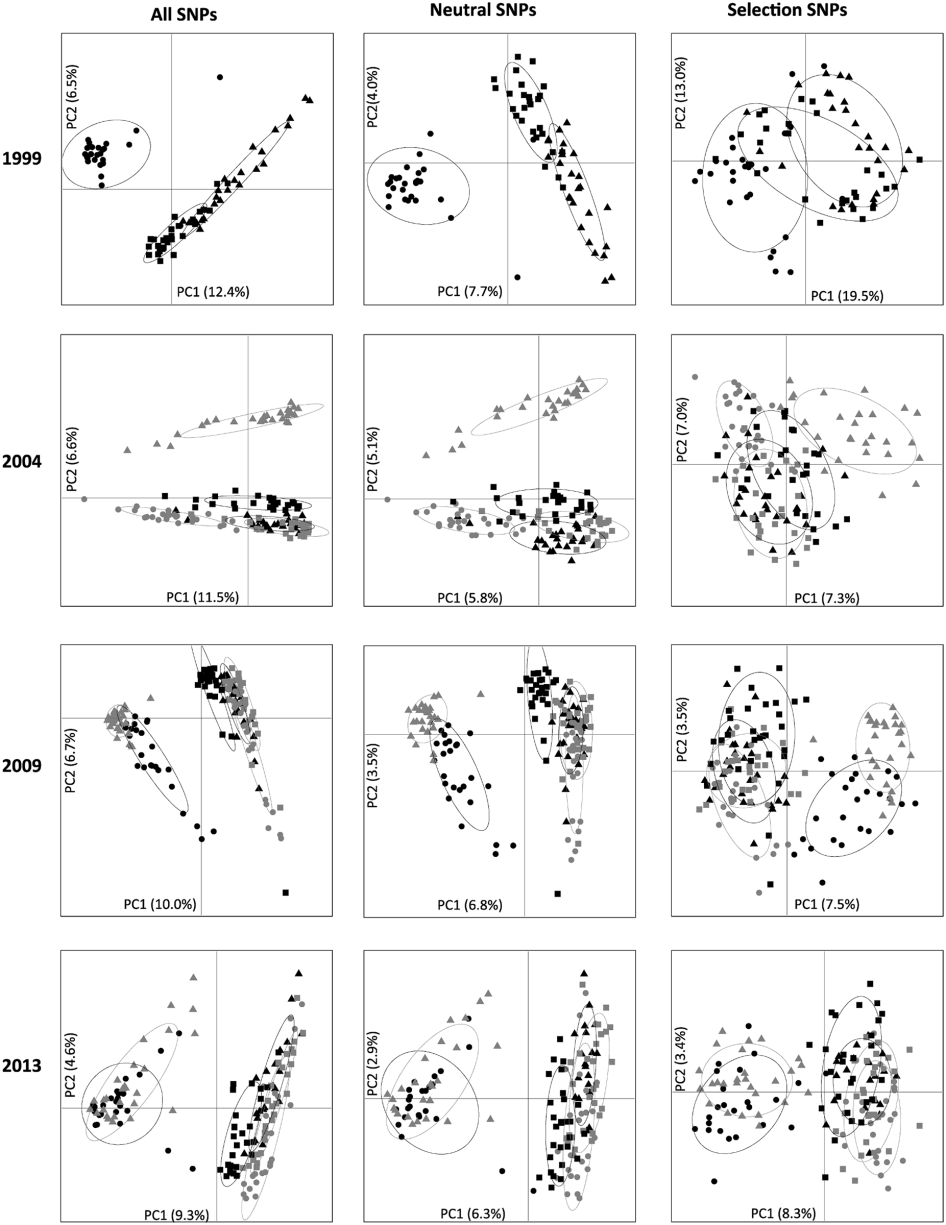
Principal component analysis (PCA) of population specific allele-frequencies for: all SNPs, neutral SNPs and SNPs under selection for each year as inferred by BAYESCAN. Populations are indicated with symbols: Arthur River (black circle), Woolnorth (grey triangle), Narawntapu (black square), Mt William (grey square), Freycinet (black triangle) and Forestier (grey circle). For each PCA, percentage of variation that the first two principal components (PC1 and PC2) explain is given.

PCA plots of the SNP frequency matrices for each sampling time show that variation is explained by the geographic distribution of the populations, regardless of whether based on ‘all SNPs’, ‘neutral SNPs’, or ‘selection SNPs’ (Fig 3). All PCA plots identify two clusters, a northwest (Woolnorth and Arthur River) and an eastern cluster (Narawntapu, Mt. William, Freycinet and Forestier). The amount of variation explained by the two major axes was low (PC1<20% and PC2<13%) (Fig 3). We found no evidence for increased differentiation among populations over time in relation to DFTD spread for the SNPs under selection and the ‘selection SNP’ pattern matched the ‘neutral SNP’ pattern (Fig 3).

### Selection—‘multiple time points’

**Fig 4:**
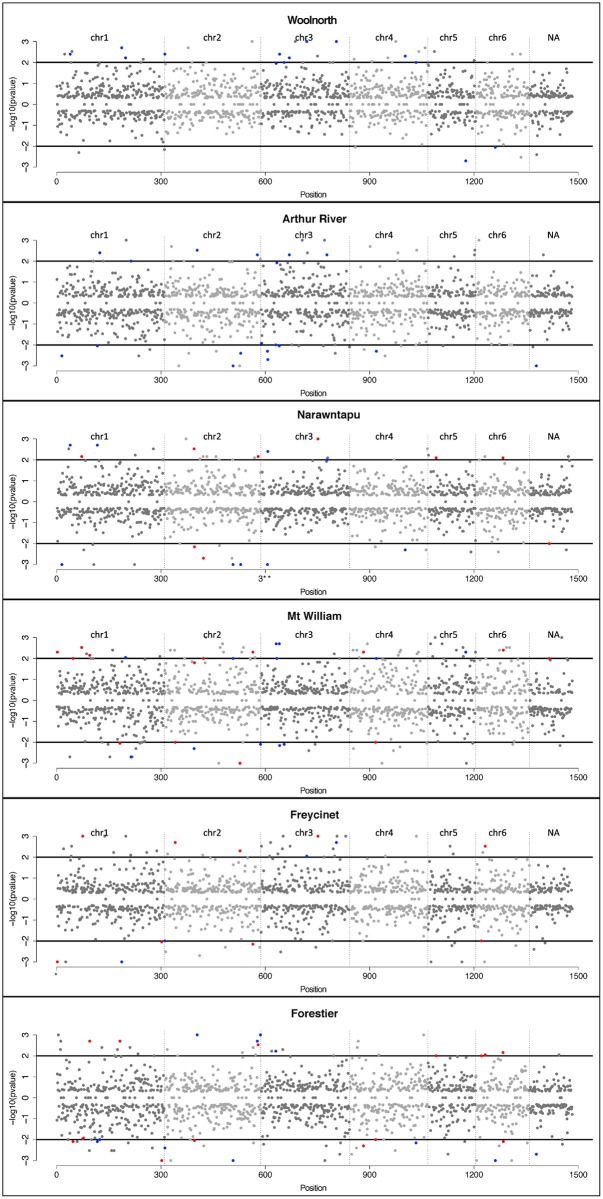
Support for SNPs under selection in Tasmanian devil populations. Results from WFABC based on an effective population size of *N*_e_ = 500. Black vertical lines divide the SNPs into chromosome assignment. For each SNP Bayesian *P*-value on a log_10_ scale is given. Black horizontal lines correspond to a threshold of *P* = 0.01 for positive selection (above) and negative selection (below) null. Loci under selection for multiple populations are indicated with colored dots: non-DFTD vs. DFTD (blue) and DFTD vs. DFTD (red); none of the SNPs were shared only between the control populations.

Loci under positive and negative selection were inferred for all populations, using a ‘multiple time points’ approach implemented in WFABC—although the majority of SNPs appeared neutral (Fig 4). In total 17 and 336 SNPs were identified as being under selection under a small (*N*_e_ = 50) and large small (*N*_e_ = 500) *N*_e_, respectively (S3 File). The SNPs under selection were distributed across the genome and no selection ‘hot–spots’ was apparent (Fig 4). A small number of SNPs under selection were shared among populations, some in the same direction (‘positive–positive’ and ‘negative–negative’) (Table 2a), and others in opposite direction (‘positive–negative’ and ‘negative–positive’) (Table 2b).

**Table 2:** Pairwise number of shared SNPs under selection in Tasmanian devil populations assuming a large effective population size (*N*_e_ = 500). The patterns are column–by–row for a) SNPs under selection in the same direction, positive–positive (above diagonal) and negative-negative (below diagonal), and b) SNPs under selection in opposite direction, positive-negative (above diagonal) and negative-positive (below diagonal).

			Site number					
			1	2	3	4	5	6
**a)**		**Positive/positive**						
	**Negative/negative**	1. Woolnorth	-	0	1	2	2	0
		2. Arthur River	0	-	2	3	0	1
		3. Narawntapu	0	0	-	1	1	3
		4. Mt William	0	2	0	-	0	1
		5. Freycinet	0	0	0	0	-	1
		6. Forestier	1	1	2	1	2	-
**b)**		**Positive/negative**						
	**Negative/positive**	1. Woolnorth	-	0	1	2	1	2
		2. Arthur River	1	-	4	0	0	1
		3. Narawntapu	0	1	-	1	0	1
		4. Mt William	0	1	3	-	2	4
		5. Freycinet	0	0	0	0	-	1
		6. Forestier	0	3	0	1	1	-

### Selection—‘single’ vs. ‘multiple time points’

Combining the results from the ‘single time point’ and ‘multiple time points’ approaches shows that BAYESCAN and WFABC only inferred two and 64 of the same SNPs to be under selection assuming small (*N*_e_ = 50) and large (*N*_e_ = 500) effective population sizes, respectively (S4 File). Furthermore, the two methods inferred that selection works in opposing direction for 1/2 of SNPs assuming a small *N*_e_ and 31/64 SNPs assuming a large *N*_e_ (S4 File). All SNPs invoked to be under selection with BAYESCAN were influenced by positive selection, whereas WFABC invoked approximately half of the SNPs to be under positive selection and half to be under negative selection. Furthermore, there was incongruence in the selection direction among populations between the two methods (Table 1).

## Discussion

Using genome-wide RAD markers for hundreds of individuals sampled across time, in multiple DFTD affected populations, and non-infected control populations, we investigated possible selective responses to DFTD, an infectious cancer causing severe declines in Tasmanian devil populations. We did not find any consistency in the loci inferred to be under selection, both among multiple populations affected by DFTD through time, or among different approaches to identify loci under selection. The majority of SNP frequency changes ascribed to selection by ‘single time point’ and ‘multiple time points’ approaches—the latter assuming large *N*_e_—are most likely ‘false positives’; underlying genetic differences are more likely a product of genetic drift rather than selection [6].

### Selective response to DFTD over time–‘single time point’ approach

A disease with 100% fatality is expected to enforce a massive selection pressure on affected populations, particularly with respect to ability to breed before mortality [27]. It is expected to cause loss of genetic diversity, either as a result of selection for specific alleles or as the result of increased genetic drift in a declining population [28]. *N*_e_ is a measure of the degree of genetic drift in a population, and the interaction between *N*_e_ and selection—described as *N*_e_ × selection coefficient (*s*)—determines the fate of an allele [29]. An allele under selection will be more likely go to fixation when either *N*_e_ or *s* are very large. In most endangered wildlife the former requirement is not met [30], thus selection will be of lesser importance than genetic drift for shaping genetic diversity in the population. Tasmanian devil populations with and without DFTD all had similar genetic diversity—a pattern that remained unaffected over time in DFTD populations (S1 File). The already low genetic diversity in devils—a result of extensive past population declines [22]—might have constrained DFTD from causing further decline in genetic diversity, particularly if the genetic diversity immediately prior to DFTD emergence was not at equilibrium with population size.

Over the 15 year sampling period (1999 to 2013), DFTD entered three of the six populations under investigation (Fig 1a). Despite extensive population declines in DFTD affected populations and a potentially very high selection pressure, no consistent selection pattern emerged from the ‘single time point’ approach. If there was strong selection for particular SNPs in this system, over time selection coefficients (*s*) would lead to an increase in differentiation on the PCA of SNPs under selection as DFTD entered Freycinet (2001), Forestier (2004) and Narawntapu (2008) (Fig 3). The lack of a selective response is either due to weak *s*, small *N*_e_ or a combination of the two [29]. One could argue that the period of time under investigation in this study (15 years, corresponding to 7–15 generations depending on the extent of precocial breeding) was too short for detecting loci under selection. If this were the case, one would expect BAYESCAN to detect a consistent set of loci to be under selection in each population throughout the study (1999 through to 2013)—a pattern that we did not observe. That the number of loci under selection increased over time only reflects the increasing number of populations analyzed during these later periods (SNPs_1999_ = 20 in 3 populations; SNPs_2004_ = 55 in 5 populations; SNPs_2009_ = 141 in 6 populations; SNPs_2013_ = 128 in 6 populations;) (S2 File), and suggests that ‘single time point’ approaches are prone to ‘false positives’ [5]. The inclusion of ‘minor allele frequency’ (MAF) loci in the analysis could bias the number of false–positives upward [5, 31], but as our dataset contained no MAFs (<0.05) this bias is unlikely to have influenced our results. Our results highlight that caution should be taken before invoking selection, and basing conservation measures on these inferences, from ‘single time point’ approaches. Combining multiple ‘single time point’ approaches—as we have done here—offers a way to increase robustness of interpretation.

### Selective response in DFTD and non-DFTD affected populations–‘multiple time points’ approach

The small *N*_e_ of the devil populations [19, 22] constrains the potential for a selective response to DFTD. Only when we assume an *N*_e_ that is unrealistically large do we infer a larger number of loci to be under selection (Fig 4; S3 File), but inconsistent patterns in the direction of selection emerge, suggesting that ‘false positives’ increase proportionally with assumed *N*_e_ (S3 File). A strong selective response to DFTD would show a consistent directional selection pattern—positive or negative—across DFTD affected populations [10]. We would expect to consistently identify these SNPs with a ‘multiple time points’ approach by tracing allele frequencies over time in multiple DFTD affected populations. The results when assuming a small *N*_e_ (*N*_e_ = 50), where few loci are inferred to be under selection (Table 1b; S3 File), are likely a better representation of the situation in Devil populations. The pattern is inconsistent across populations, but fits better with what we would expect given the established small *N*_e_. WFABC is a relatively new method and it would be useful for future studies to assess its performance under different levels of genetic diversity, population structure, selection pressures, and *N*_e_.

### Incongruence between ‘single time point’ and ‘multiple time points’ approaches

It is interesting that the two methods for inferring loci under selection give different results, both with regards to which SNPs are under selection (S2 File; S3 File) and the direction of selection (Table 1; S4 File). Whereas the ‘multiple time points’ approach (WFABC) invoked SNPs to be under both positive and negative selection, our ‘single time point‘ approach (BAYESCAN) only inferred positive selection. BAYESCAN uses a single sample of multiple populations to detect outliers based on differences in *F*_ST_ while accounting for population structure and differences in ancestral allele sharing [5]. BAYESCAN generally performs well for varying selection across clinal environments, but is not very efficient under the isolation-with-migration model [32]. WFABC on the other hand uses temporal sampling of individual populations combined with an ABC approach, and simulates a large number of datasets under user specified parameters [8, 9]. The tracing of allele frequencies over time might make WFABC sensitive to population structure; this stands to be tested. Our study highlights the importance of not relying on only one method—‘single time point’ or ‘multiple time points’—when investigating loci under selection, but to instead apply methods with different performance optima (e.g. accounting for population structure and background selection,) and assess congruence of results. Analysis of replicates (e.g. several populations) also enables the detection of incongruence in the results, and should be strongly encouraged. Development of methods that incorporate a ‘multiple time points’ approach while accounting for population structure would be useful.

### Future for the Tasmanian devil

Previous analyses of the effects of DFTD on genetic diversity used ten microsatellite markers to investigate selection. One locus was identified as being under selection associated with DFTD [18], which is likely an artifact of the selection method not accounting for demographic history [19, 33]. Although the ~1,500 SNPs used in this study are spread across the genome, the low genetic diversity of Tasmanian devils [34] might hamper the chance of one of these SNPs being in close proximity to a gene under selection [35]. If there is directional selection, it is likely to be quite localized in the genome. A fully annotated genome and information on LD across the genome, combined with a very large number of SNPs or a targeted gene approach, might yet allow identification of alleles associated with DFTD driven selective response. Potentially the precocial breeding could be driven by sex-linked loci [36]. Taken together these results suggest that past bottlenecks severely decreased the species genetic diversity, resulting in effective population sizes of the Tasmanian devil that are too small for allele frequencies to be easily driven by selection. Low genetic diversity is tightly linked to increased risk of decreased fitness [37], highlighting the need for conservation measures—aiming at conserving genetic diversity—in order to assure that the Tasmanian devil’s evolutionary potential does not get further depleted.

## Materials and Methods

### Sample collection and DNA extraction

Ear biopsies from Tasmanian devils (*n* = 527) were collected in 1999–2013 under the approval of the University of Tasmania Animal Ethics Committee (statements A0008588, A0010296, A0011696, and A0013326). Samples were collected under the authority of the Department of Primary Industries, Parks. Water, and Environment, Tasmanian Government. The samples were from six locations representing the species’ geographic range (Fig 1a). DFTD sites (with DFTD arrival in parenthesis) are represented by: Mt. William (1996), Freycinet (2001), Forestier (2004) and Narawntapu (2008). DFTD-free control sites are Woolnorth and Arthur River. Genomic DNA was extracted using the Agencourt DNAdvance kit (Beckman Coulter). Standard protocol was followed except that 50% more Proteinase K was used to aid digestion. DNA concentration was quantified using a Fragment Analyzer between 10–30 kbp. Approximately 5 ng DNA was used for RAD sequencing.

### RAD–tag genotyping and assignment of tags to chromosomes

Each sample was genotyped using Nextera–fragmented, reductively amplified DNA (nextRAD). Genotyping by sequencing was carried out using nextRAD markers (libraries prepared and sequenced by SNPsaurus, LLC). Each read was 100 bp in length. A reference set of loci was defined corresponding to haplotypes having ≥2000 reads across the population. Haplotypes were collapsed by alignment and the single haplotype with most reads was set as the reference for that locus. Reads were aligned to the reference and for each locus haplotypes present at ≥10% of the samples and ≥4% of the reads were retained. For each sample error correction/imputation was used to assign as many reads as possible to haplotypes on a sample-by-sample basis. For a haplotype to be included in the dataset it had to be present in at least 1/12th of the reads of a individual to be counted. Each locus in each individual was checked for ploidy, and loci with >2 haplotypes for a given individual were excluded. Loci with <6 reads in an individual were coded as missing genotypes. The RAD fragments were aligned to the devil reference genome (GenBank: GCA_000189315.1) and divided into chromosomes (1–6 and X; Y chromosome it not part of the reference genome). As the Tasmanian devil genome is assembled to scaffold level, we could assign each RAD read to a chromosome, but not map them more specifically. To assure independence of SNP loci only one SNP—chosen randomly—per RAD fragment was included.

### Genetic diversity in DFTD and non–DFTD populations

Because we were interested in tracing changes in population genetic parameters over time, and their possible correlation with DFTD spread, we partitioned our data into annual samples (1999, 2004, 2009 and 2013). For each sample we investigated temporal and spatial changes in genetic diversity. We used GENALEX v6.5 [38] to calculate the mean number of alleles per SNP (*A*), number of private alleles (*A*_p_), observed heterozygosity (*H*_O_), and expected heterozygosity (*H*_E_). For this analysis we included all autosomal SNPs (*n* = 1359) and SNPs with unknown chromosome location (*n* = 123). Some of the SNPs with unknown chromosome location could potentially be located on the X chromosome, but given the low number we expect potential bias associated with these in the downstream analyses to be minor. To test for temporal differences in genetic diversity we used linear regression analysis with Student’s *t*-test and Fisher’s *F*-test to assess significance [39].

### Selection—‘single time point’

To infer loci under selection at each of the four sampling periods (1999, 2004, 2009 and 2013) we used BAYESCAN v2.1 [5]. This ‘single time point’ approach scans for loci under selection by decomposing *F*_ST_ into a population-specific component *β*, and a locus-specific component *α*, that are sensitive to the strength of selection on individual loci. For each locus the posterior probability is estimated for a neutral model (*α* = 0) and a model including selection (*α*≠0), for positive (α>0) and negative (*α*<0) selection, respectively. The best–fit model is identified based on posterior odds ratios (PO) for the model of local adaptation relative to the neutral model.

We used prior odds of 10, corresponding to a prior belief that the neutral model is 10 times more likely than the model including selection. ‘False positive rates’ decrease with increasing prior odds [33], but as we were interested in investigating possible overlap in detection of SNPs under selection with BAYESCAN and the ‘multiple time points’ approach—WFABC [9]—we used ‘vague’ (1:10) prior odds. Posterior distributions of degree of differentiation (*β* and *α*) were obtained using Reversible-Jump MCMC. We tested each sample separately (1999, 2004, 2009 and 2013) using sample size = 5×10^3^, thinning interval = 10^2^, pilot runs = 10^2^, pilot run length = 10^4^, and additional burn-in = 5×10^5^. Convergence was assessed in R v3.0.2 [40] using the CODA package following Geweke’s [41] convergence diagnostics by comparing the mean of the first 10% of the MCMC chain with the mean of the last 50% after removal of the burn-in. The output was plotted in R to identify outliers with a ‘false positive rate’ of maximum 5%.

To investigate the effect of the prior odds we also ran the analyses with prior odds of 100 corresponding to the neutral model being 100 times more likely than the selection model (S5 File). This decreased the number of loci under selection, but as we are interested in overlap in outliers between the ‘single time point’ and multiple time points’ approach, we considered the analyses with prior odds of 10 in the following. We only considered SNPs to be under selection if they fell into the following categories of support: ‘strong’ (BF = 10–32), ‘very strong’ (BF = 32–100), and ‘decisive’ (BF = 100–∞). For each class of SNPs (all, neutral and positive selection) we made a principal component analysis (PCA) plot in R using the ADEGENET v1.4–2 package [42].

### Selection—‘multiple time points’

To infer loci affected by selection in each population (Woolnorth, Arthur River, Narawntapu, Mt. William, Freycinet and Forestier) over time we used WFABC v1.0 [8, 9]. This approach is based on a Wright-Fisher model to infer selection coefficients (*s*) from time-sampled populations. All loci share the same *N*_e_ but have individual *s*. An Approximate Bayesian Computation (ABC) approach is used to simulate datasets for the *s* trajectories for each locus. We fixed *N*_e_ for each population—as the effective population size of devils appears unaffected by DFTD [19]. Loci under selection are identified based on Bayesian ‘*P*-values‘ corresponding to *P*(*s<*0|*x*), where a locus is considered to be under selection if its equal-tailed 100(1–*P*)% posterior density distribution excludes zero [1].

We used fixed effective population sizes of *N*_e_ = 50 corresponding to lower previous estimates [19], and investigated the effects of increasing *N*_e_—thereby reducing the effects of drift relative to selection—to the maximum estimated *N*_e_ for the entire devil population (*N*_e_ = 500) [22]. We used 10^5^ bootstrap replicates and broad uniform priors for *s* (-1.0–1.0) to infer loci under selection. The generation time for Tasmanian devils is 2 years [43]. Populations affected by DFTD show precocial mating leading to a decrease in generation time [17]. We analyzed the dataset with a conservative approach—assuming a generation time of 2 years for all populations—and explored the effect of decreasing the generation time to 1 year for the populations affected by DFTD. As expected this only increased the rate of change in allele frequencies over time in the DFTD populations (data not shown).

## Supporting Information

S1 File Estimates of genetic diversity for the Tasmanian devil based on 1482 SNPs. (PDF)

S2 File SNPs under selection at each year detected with BAYESCAN assuming prior odds of 10. (PDF)

S3 File SNPs under selection in individual Tasmanian devil population detected with WFABC assuming a) a small (*N*_e_ = 50) and b) a large (*N*_e_ = 500) effective population size. (PDF)

S4 File Overview of SNPs under selection detected with demographic and time-series methods. (PDF)

S5 File SNPs under selection at each year detected with BAYESCAN assuming prior odds of 100. (PDF)
