# Supplementary material for: Detecting Selection on Temporal and Spatial Scales: A Genomic Time-Series Assessment of Selective Responses to Devil Facial Tumor Disease
Source: PLoS One. 2016 Mar 1;11(3):e0147875. doi: 10.1371/journal.pone.0147875 (PMC4773136; doi:10.1371/journal.pone.0147875)
Supplement: S1 File — (PDF) [file pone.0147875.s001.pdf]

SI 1. Estimates of genetic diversity for the Tasmanian devil based on 1482 SNPs. The statistics correspond to: number of individuals genotyped (N), mean number of alleles per SNP (A), number of private alleles (A_p), observed heterozygosity (H_O), and expected heterozygosity (H_E). Presence and absence of devil facial tumor disease (DFTD) is indicated with plus and minus symbols, respectively.

**Population**	**Year**	**DFTD**	N	A	A_p	H_O	H_E
Woolnorth	2004	-	27	1.88	11	0.408	0.315
	2009	-	27	1.91	-	0.408	0.315
	2013	-	27	1.93*	-	0.425	0.336
Arthur River	1999	-	27	1.94	13	0.425	0.327
	2009	-	25	1.93	-	0.400	0.326
	2013	-	24	1.93	-	0.393*	0.317
Narawntapu	1999	-	27	1.98	7	0.439	0.352
	2004	-	26	1.97	-	0.442	0.351
	2009	+	30	1.98	-	0.450	0.352
	2013	+	27	1.97	1	0.440	0.356
Mt William	2004	+	27	1.96	-	0.426	0.333
	2009	+	26	1.95	-	0.440	0.344
	2013	+	27	1.95	-	0.404	0.332
Freycinet	1999	-	26	1.96	8	0.452	0.352
	2004	+	27	1.96	-	0.446	0.345
	2009	+	27	1.97	-	0.439	0.352
	2013	+	20	1.94	1	0.434	0.348
Forestier	2004	-	26	1.91	-	0.443	0.341
	2009	+	24	1.94	-	0.455	0.348
	2013	+	26	1.93	-	0.393	0.328

Significant change over time is indicated with * (P<0.05).
