# Supplementary material for: Detecting Selection on Temporal and Spatial Scales: A Genomic Time-Series Assessment of Selective Responses to Devil Facial Tumor Disease
Source: PLoS One. 2016 Mar 1;11(3):e0147875. doi: 10.1371/journal.pone.0147875 (PMC4773136; doi:10.1371/journal.pone.0147875)
Supplement: S2 File — (PDF) [file pone.0147875.s002.pdf]

SI 2. SNPs under selection at each year detected with BAYESCAN assuming prior odds of 10. For each SNP chromosome (chr.), super-contig, SNP position in super-contig and direction of selection, which for all SNPs are positive (P).

Chr.	Super-contig	SNP position	1999	2004	2009	2013
1	2	960133	-	P	P	-
1	5	3478545	-	P	-	P
1	12	876195	-	P	P	-
1	24	2439039	-	-	-	P
1	30	456063	-	-	-	P
1	34	1974145	-	-	P	-
1	34	2090729	-	-	P	-
1	40	2957400	-	-	P	P
1	50	417458	-	-	P	P
1	54	1481620	-	-	-	P
1	89	1980127	P	P	P	-
1	103	63790	-	-	P	P
1	107	470615	-	-	-	P
1	109	440318	-	-	P	-
1	109	452958	-	-	P	-
1	124	537278	-	-	-	P
1	130	1460525	-	-	P	P
1	174	250444	-	-	-	P
1	212	560709	-	-	P	P
1	239	83999	-	-	-	P
1	239	2175315	-	-	P	P
1	259	53959	P	-	P	P
1	259	73417	P	-		P
1	259	2775110	P	P	P	P
1	281	786550	P	P	-	P
1	281	1194959	P	P	-	P
1	289	1692290	P	P	P	P
1	297	1446547	-	-	-	P
1	354	2358931	-	-	-	P
1	375	696764	-	-	P	P
1	381	278795	-	-	-	P
1	386	537974	-	-	P	P
1	397	80203	-	-	P	P
1	445	71025	-	P	P	P
1	445	75680	-	P	P	P
1	446	132042	-	-	-	P
1	446	133260	-	-	-	P
1	630	3069	P	P	P	P
1	643	103647	-	P	P	P
1	788	56371	-	-	-	P
1	1074	6834	-	-	-	P
1	1079	4982	-	-	P	P
1	1246	33567	-	-	P	P
1	1301	46853	-	-	-	P
1	1370	3906	-	-	P	P
1	1431	18549	-	P	-	-
1	1503	12070	-	-	P	-
1	3080	6092	-	-	P	-
1	5302	2045	-	-	P	P
2	13	8347	-	P	P	P
2	67	673351	-	-	P	-
2	117	748966	-	-	-	P
2	122	525086	-	-	P	-

2	154	3701007	-	-	P	P
2	185	258819	-	-	P	-
2	223	552281	-	-	-	P
2	231	725912	-	-	-	P
2	233	676222	-	-	P	-
2	235	2941774	-	-	P	P
2	241	412933	-	P	P	P
2	253	2410314	-	-	P	P
2	254	173178	-	-	P	P
2	256	209544	-	-	P	-
2	279	1962190	P	-	-	-
2	310	225423	-	P	-	-
2	310	2192602	-	-	P	-
2	313	1149368	-	-	P	-
2	382	421209	-	-	P	P
2	445	3244531	-	-	P	P
2	447	35597	-	-	P	-
2	447	167640	-	-	P	-
2	449	170227	-	-	P	P
2	451	570671	-	-	P	-
2	458	456749	-	-	-	P
2	462	195643	-	-	P	P
2	569	60916	-	P	P	P
2	658	62646	-	-	P	P
2	1150	21386	-	-	-	P
2	1421	58284	-	-	P	P
2	2426	18973	-	-	P	-
3	93	249731	P	P	P	P
3	94	718106	P	P	P	P
3	94	779170	P	-	P	P
3	109	1314065	-	P	P	P
3	109	1363279	-	P	-	-
3	137	836212	-	-	P	-
3	184	863591	-	-	P	P
3	195	3570500	-	-	P	-
3	243	2485658	-	-	P	-
3	245	93639	-	-	P	P
3	260	2537254	-	-	P	-
3	268	1240699	-	-	P	-
3	310	1770399	-	-	P	P
3	314	2784883	-	-	P	-
3	320	3690596	P	-		-
3	330	833277	-	-	P	-
3	342	355159	-	-	P	-
3	354	1712873	-	-	P	P
3	357	217434	-	-	P	-
3	377	2417877	-	-	P	P
3	381	4795550	-	P	-	-
3	387	2836116	-	-	P	P
3	388	1859272	-	-	P	-
3	388	3437445	-	P	P	P
3	390	2536138	P	-	P	P
3	450	160463	-	P	-	-
3	663	8767	-	-	P	P
3	760	37586	P	P	P	P
3	1425	53040	-	-	P	-
3	1483	4552	-	-	P	-
3	3680	908	-	P	P	P
3	7012	906	-	-	P	-
4	4	597847	-	-	P	-

4	14	426490	-	P	P	P
4	15	1005633	-	-	P	P
4	15	1523425	-	-	P	P
4	17	3632032	-	P	P	-
4	23	331080	-	-	-	P
4	30	2804952	-	-	P	-
4	32	54662	-	P	P	P
4	60	1569690	-	-	-	P
4	61	345278	-	P	P	P
4	77	2113411	-	P	P	P
4	87	1670726	-	-	P	P
4	102	1524824	-	P	-	-
4	102	1561669	-	-	P	-
4	103	1119812	-	-	P	-
4	103	1133697	-	P	P	-
4	103	1139356	-	-	-	P
4	127	1864609	P	-	-	-
4	130	369461	-	-	P	-
4	155	685114	-	-	-	P
4	175	668147	-	-	-	P
4	181	101628	-	P	P	-
4	207	1303526	-	-	P	P
4	237	956702	-	-	P	-
4	250	2091405	-	P	P	P
4	251	828774	-	P	-	-
4	274	179109	-	-	P	P
4	276	1861899	-	P	P	P
4	281	706685	-	-	P	-
4	298	71438	-	-	P	P
4	302	548044	-	P	P	P
4	463	41732	-	-	P	P
4	657	33346	-	-	P	P
4	657	127489	-	-	P	P
4	887	27413	-	P	-	-
4	1078	47296	-	-	-	P
4	1129	37547	-	P	-	-
4	1135	20117	-	-	-	P
4	1826	196	-	P	-	P
4	1885	1624	-	-	P	-
5	10	76100	P	-	P	P
5	43	1194068	-	-	P	-
5	50	1376047	-	-	-	P
5	55	1312293	-	-	P	P
5	63	1205307	-	-	-	P
5	68	250972	-	P	P	P
5	82	124066	-	P	P	P
5	82	1023371	-	-	-	P
5	85	2268240	-	-	P	-
5	119	4088160	-	-	P	-
5	157	1444854	-	P	P	P
5	170	2852763	-	-	P	P
5	175	242072	-	-	P	P
5	182	227713	-	P	P	P
5	368	180120	-	-	P	-
5	374	40069	-	-	P	P
5	379	97233	-	-	P	P
5	447	7205	-	-	P	P
5	460	53554	-	-	P	P
5	464	46402	-	-	P	-
5	523	19999	-	-	-	P

5	625	3774	P	P	P	P
5	632	10345	-	P	P	-
6	41	225810	-	-	-	P
6	82	187751	-	-	P	P
6	83	554595	-	P	-	-
6	94	165143	-	-	P	P
6	106	775062	-	-	-	P
6	121	808041	-	-	P	P
6	140	3332564	-	-	P	-
6	145	2409985	-	P	-	P
6	155	209370	-	P	-	-
6	156	2519435	-	-	P	P
6	161	1965251	-	-	P	P
6	167	3269766	-	-	P	P
6	168	299080	-	P	-	-
6	168	390402	-	-	P	P
6	191	49380	-	-	-	P
6	313	113496	-	-	-	P
6	425	5868	-	-	P	-
6	728	9316	-	-	P	-
NA	0	2	P	P	P	P
NA	0	4	-	P	-	-
NA	0	12	P	-	-	-
NA	0	18	-	P	P	P
NA	0	34	-	-	P	-
NA	0	47	-	P	-	-
NA	0	91	-	-	P	P
