# Supplementary material for: Detecting Selection on Temporal and Spatial Scales: A Genomic Time-Series Assessment of Selective Responses to Devil Facial Tumor Disease
Source: PLoS One. 2016 Mar 1;11(3):e0147875. doi: 10.1371/journal.pone.0147875 (PMC4773136; doi:10.1371/journal.pone.0147875)
Supplement: S3 File — (PDF) [file pone.0147875.s003.pdf]

SI 3. SNPs under selection in individual Tasmanian devil population detected with WFABC assuming a) a small (N_e=50) and b) a large (N_e=500) effective population size. For each SNP chromosome (Chr.), super-contig and position in super-contig are given. Direction of selection is given as positive (P) or negative (N).

	**Chr.**	**Super-contig**	**SNP position**	**Arthur River**	**Woolnorth**	**Narawntapu**	**Mt William**	**Freycinet**	**Forestier**
a)									
	1	301	3268273	-	-	-	-	P	-
	1	301	3286057	-	-	-	-	P	-
	2	42	2706312	-	-	-	-	-	P
	2	67	673351	-	-	N	-	-	N
	2	333	1747729	P	-	-	-	-	-
	2	334	978337	-	-	-	P	-	-
	2	343	1707547	-	-	-	P	-	-
	3	35	1680796	-	-	-	-	P	-
	3	104	4367326	-	-	-	-	P	-
	3	126	2077698	-	P	-	-	-	-
	3	357	217434	-	P	-	-	-	-
	3	1483	4552	N	-	-	-	-	-
	4	127	1864609	-	-	-	-	P	-
	5	43	1194068	-	-	-	P	-	-
	5	507	10806	-	-	-	-	N	-
	6	130	148254	-	-	-	-	-	N
	6	158	1961285	-	-	-	-	-	N
b)									
	1	2	960133	-	-	-	P	-	-
	1	4	146619	N	-	-	-	-	-
	1	5	3478545	-	-	-	P	-	P
	1	11	2400455	-	-	-	P	-	-
	1	12	876195	N	-	-	-	-	-
	1	14	70407	-	-	-	-	-	P
	1	24	1707150	-	-	-	P	N	-
	1	31	111314	P	-	N	-	-	-
	1	34	2090729	-	-	-	N	-	-
	1	34	2451414	-	-	P	-	-	-
	1	40	649982	-	-	-	-	-	N
	1	40	3849632	P	-	-	-	-	-
	1	49	312113	-	-	-	N	-	-
	1	51	1401829	-	-	-	P	-	N
	1	53	3718041	N	-	-	N	-	-
	1	54	1481620	-	-	-	-	N	-
	1	72	1595488	-	-	-	-	P	-
	1	78	341120	-	P	-	-	-	-
	1	78	1218118	-	P	-	P	-	-
	1	95	249851	-	P	-	-	-	-
	1	96	1372113	-	-	-	P	-	-
	1	124	537278	-	P	-	-	N	-
	1	124	692378	-	P	-	-	-	-
	1	128	3859380	-	-	-	-	-	P
	1	191	2271354	-	-	-	-	N	N
	1	215	1505212	-	-	-	-	-	P

1	216	2220684	-	-	P	-	-	-
1	218	379502	-	P	-	-	-	-
1	233	4202709	-	-	-	P	-	-
1	238	382160	-	-	P	-	-	-
1	258	338961	-	-	-	-	-	N
1	259	73417	-	-	-	-	-	P
1	259	2775110	-	-	-	-	P	-
1	268	1684946	-	-	-	-	-	P
1	270	365503	-	N	-	-	-	-
1	273	222873	-	-	-	P	-	-
1	276	962756	-	-	P	P	-	-
1	289	1692290	-	-	N	-	-	-
1	297	1446547	-	-	N	-	-	-
1	298	1303323	-	-	-	-	P	-
1	301	3268273	-	-	-	-	P	-
1	301	3286057	-	-	-	-	P	N
1	304	1251512	-	-	-	-	P	-
1	312	562647	P	-	P	-	-	N
1	317	226196	-	-	-	-	-	P
1	324	80765	-	-	-	N	-	-
1	324	768688	-	-	-	N	-	-
1	332	827052	-	-	-	N	-	-
1	332	970742	-	-	-	N	-	-
1	349	890585	-	-	-	-	-	N
1	350	40076	-	-	P	-	-	-
1	363	50875	-	-	-	-	P	-
1	367	342318	-	N	-	-	-	-
1	370	1478384	-	-	-	N	-	-
1	375	696764	N	-	-	-	-	N
1	381	278795	-	-	-	P	-	-
1	381	1439904	-	-	-	-	-	P
1	382	886856	P	-	-	-	-	-
1	382	1574864	-	-	-	-	-	N
1	396	929366	-	-	N	-	-	-
1	437	201196	-	-	-	-	-	N
1	444	198	-	-	-	-	-	P
1	445	71025	-	-	-	-	-	N
1	445	75680	-	-	-	-	-	N
1	446	132042	-	-	-	N	-	-
1	446	133260	-	-	-	N	-	-
1	447	107385	-	-	-	-	-	N
1	491	2551	-	-	N	-	-	-
1	619	67319	-	-	-	-	-	P
1	630	3069	N	-	-	-	-	-
1	638	61005	-	P	-	-	-	-
1	643	103647	-	-	-	N	-	-
1	657	108426	-	-	-	-	P	-
1	718	7366	-	-	-	-	P	-
1	788	56371	-	-	-	-	-	P
1	878	4969	-	P	-	-	-	-
1	1143	15801	-	-	P	-	-	-
1	1217	1507	-	-	-	N	-	P
1	1246	33567	-	-	P	-	-	-
1	1370	3906	-	-	-	-	P	-
1	3418	15084	-	-	-	-	-	P
1	4828	268	-	P	P	-	-	-
2	42	2706312	N	-	-	-	-	P
2	51	134372	-	-	-	P	-	-
2	59	3480458	-	-	P	-	-	-
2	62	242764	-	-	-	-	N	-

2	67	673351	P	-	N	P	-	N
2	75	54057	-	P	-	-	-	-
2	102	195820	-	-	-	-	-	P
2	112	797257	P	-	-	-	-	-
2	122	525086	-	-	N	-	-	-
2	139	762099	N	-	-	-	-	-
2	162	1342117	-	-	-	-	-	N
2	175	2107192	-	P	-	-	-	-
2	177	1882795	-	-	-	-	P	-
2	182	925721	-	-	P	-	-	-
2	185	258819	-	-	P	-	-	-
2	205	2650157	-	-	N	-	-	-
2	214	2112250	-	-	-	P	-	-
2	233	676222	-	-	P	-	-	-
2	233	1387404	-	-	-	N	P	-
2	235	2941774	-	-	P	-	-	-
2	254	173178	-	-	P	-	-	P
2	255	791720	-	-	-	-	-	P
2	256	209544	-	P	-	-	-	-
2	256	4224366	-	-	-	N	P	-
2	260	1144836	N	-	-	-	-	-
2	263	147829	-	-	-	-	N	-
2	269	89605	-	-	-	-	-	P
2	271	1507386	P	-	-	-	-	-
2	279	1962190	-	-	N	-	-	-
2	282	1529946	N	-	-	-	-	P
2	292	3561006	-	-	-	N	-	-
2	313	1149368	-	-	-	-	-	N
2	315	139271	-	-	-	P	-	-
2	315	189600	-	-	-	P	-	-
2	333	1747729	P	-	-	-	-	-
2	334	978337	-	-	N	P	-	-
2	343	1707547	-	-	-	P	-	-
2	358	163227	-	-	-	-	-	P
2	361	618374	-	-	P	-	-	-
2	386	853403	-	-	-	P	-	-
2	398	2256226	-	-	P	N	-	-
2	408	545286	-	-	-	P	-	-
2	412	318329	-	-	-	-	P	-
2	414	677736	-	-	-	P	-	-
2	414	1245535	-	P	-	-	-	-
2	414	1462625	-	-	-	P	-	-
2	430	1249967	-	-	-	N	-	-
2	431	784343	-	-	-	-	-	N
2	439	81013	-	P	-	-	N	N
2	445	449724	-	-	-	-	-	P
2	447	118956	-	-	-	-	P	-
2	447	730861	P	-	-	-	-	-
2	448	4027821	-	-	-	P	N	-
2	451	570671	-	-	-	-	-	N
2	452	4558023	-	-	-	-	N	-
2	458	305322	P	-	N	-	-	-
2	459	1282391	N	-	-	-	-	-
2	490	82552	-	-	-	P	-	-
2	870	37273	N	-	-	N	-	P
2	919	193382	N	-	-	-	-	-
2	1078	1570	-	-	-	-	N	-
2	1164	81127	-	-	-	P	-	-
2	1486	83546	-	-	-	-	P	-
2	1762	17787	-	-	-	P	-	-

2	1854	37860	-	-	P	-	-	-
2	3023	6360	N	-	-	-	-	-
2	6746	1020	-	-	N	-	-	N
2	8034	13132	-	-	P	-	-	-
3	0	841465	N	P	-	-	-	-
3	35	1680796	-	-	-	-	P	-
3	61	1464174	-	-	P	-	P	-
3	93	249731	-	-	-	-	P	-
3	104	4367326	-	-	-	-	P	-
3	109	1363279	-	-	P	-	-	-
3	111	2291957	N	-	-	-	-	-
3	126	2077698	-	P	-	-	P	-
3	126	2119011	-	P	-	-	P	-
3	137	836212	-	P	-	-	-	-
3	138	994010	-	-	-	P	-	-
3	146	1098321	P	-	-	-	-	P
3	154	2242952	-	-	-	-	-	P
3	161	889864	-	-	-	-	-	N
3	170	861681	-	-	P	-	-	-
3	174	85819	P	-	N	-	-	-
3	197	3697299	-	-	-	-	-	P
3	199	8723	-	-	-	-	N	-
3	217	1251736	-	P	-	-	-	-
3	222	155102	-	P	-	-	-	-
3	224	324291	-	-	-	-	-	P
3	225	37277	-	-	-	-	P	-
3	225	1348164	-	-	-	-	P	-
3	243	285139	P	-	P	-	-	-
3	260	2537254	-	-	-	-	N	-
3	261	985263	-	P	-	-	-	-
3	310	1770399	-	-	-	-	-	N
3	314	2784883	-	-	-	-	P	-
3	326	81458	N	-	-	-	-	-
3	329	1389965	N	-	-	-	-	-
3	330	1011934	N	-	P	-	-	-
3	346	2735663	-	-	-	-	P	-
3	354	1772697	N	-	-	-	-	-
3	357	217434	-	P	-	N	-	-
3	357	2393022	-	-	-	-	-	N
3	367	420865	P	-	-	P	-	-
3	371	977404	-	-	-	-	P	-
3	387	3520928	-	-	-	N	-	-
3	389	3860684	-	-	-	N	-	-
3	390	2536138	-	-	-	-	-	N
3	393	3796683	-	-	-	-	P	-
3	395	857252	P	-	-	-	-	-
3	404	114318	N	-	-	P	-	-
3	413	1777	-	P	-	-	-	-
3	443	45497	-	-	-	-	-	P
3	480	85679	N	-	-	-	-	-
3	585	540185	P	-	-	-	-	-
3	740	231703	P	-	-	-	-	-
3	741	40177	P	-	-	-	-	-
3	796	30330	-	-	-	-	P	-
3	874	2261	-	P	-	P	-	-
3	929	166870	-	-	-	-	N	-
3	965	135521	-	-	-	P	-	-
3	984	24602	-	P	-	N	-	-
3	1018	128155	P	-	-	-	-	-
3	1033	468	-	-	-	-	-	N

3	1463	763	-	-	-	-	P	-
3	1483	4552	N	-	-	-	-	-
3	1580	17821	-	-	-	-	P	-
4	0	381612	-	-	N	-	-	-
4	1	91026	N	-	-	-	-	-
4	4	405721	-	-	-	-	-	N
4	4	597847	-	-	-	N	-	-
4	5	659871	-	-	-	P	-	-
4	6	875072	N	-	-	-	-	-
4	15	1005633	-	-	-	-	-	N
4	15	1523425	-	-	-	P	-	-
4	17	2238220	-	-	-	-	-	N
4	17	3632032	-	-	-	P	-	-
4	22	123626	-	-	-	P	-	-
4	26	871344	-	P	-	-	-	N
4	27	178274	-	P	-	-	-	-
4	27	2697932	-	P	-	-	-	-
4	29	87889	-	N	-	-	-	-
4	59	21414	-	-	-	P	-	-
4	60	1569690	-	-	-	-	-	P
4	60	2705358	-	-	P	-	-	-
4	66	158371	N	-	-	-	-	-
4	77	2113411	-	P	-	-	-	-
4	80	777434	-	-	-	N	-	N
4	85	1132651	-	-	-	-	-	P
4	103	1133697	-	-	-	N	-	-
4	103	1145145	-	P	-	-	-	-
4	108	2990908	-	P	-	-	-	-
4	110	164361	-	-	-	-	-	N
4	127	1864609	-	-	-	-	P	-
4	145	973634	N	-	-	-	-	-
4	145	1159821	-	-	-	P	-	-
4	155	685114	-	P	N	-	-	-
4	175	668147	-	-	-	-	P	-
4	228	4187183	-	P	-	-	-	-
4	232	58588	-	-	-	-	-	P
4	244	1367124	-	-	-	P	-	-
4	251	828774	-	-	-	-	N	-
4	251	909279	N	-	-	-	-	-
4	273	526260	-	-	P	-	-	-
4	281	4624964	-	-	-	P	-	-
4	288	1286334	P	-	-	-	-	-
4	290	184903	P	-	-	-	-	-
4	290	278523	-	P	-	-	-	-
4	293	608201	P	-	-	-	-	-
4	298	71438	-	-	N	-	-	-
4	479	48398	-	-	-	P	-	-
4	525	6048	-	-	-	N	-	-
4	541	12711	-	-	-	P	-	N
4	657	33346	-	-	-	N	-	-
4	657	127489	-	-	-	N	-	-
4	845	12122	-	-	-	-	-	P
4	902	32609	P	-	-	P	-	-
4	1826	196	-	-	-	P	-	-
5	5	381276	-	-	-	-	P	-
5	20	384808	P	-	-	-	-	-
5	42	309633	-	-	-	P	-	-
5	43	386379	-	-	-	P	-	-
5	43	1194068	-	-	-	P	-	-
5	46	1276171	-	-	P	-	-	-

5	50	1376047	-	-	-	-	P	-
5	63	1205307	-	-	-	-	-	N
5	76	2235181	N	-	-	-	-	-
5	76	2336491	-	-	-	P	-	-
5	110	4302709	N	-	-	-	-	-
5	118	1052684	-	-	-	-	N	-
5	124	263439	-	-	-	P	-	-
5	129	3511565	N	-	-	-	-	-
5	130	255772	-	-	-	P	-	-
5	144	18312	-	N	-	P	-	-
5	146	1157296	-	-	-	-	N	-
5	152	2544045	-	-	P	-	-	P
5	152	2986480	-	-	-	P	-	-
5	164	279635	-	-	-	N	-	-
5	169	1680844	-	P	-	-	-	-
5	170	174412	-	-	-	P	-	-
5	184	187135	-	-	-	N	-	-
5	318	91198	-	-	-	N	-	-
5	464	46402	-	-	-	P	-	-
5	507	10806	-	-	-	-	N	-
5	556	137196	-	-	N	-	-	-
5	600	77260	-	P	-	-	-	-
5	647	62985	-	-	-	-	-	N
5	1030	2681	-	-	-	-	-	P
5	2760	5241	-	-	-	-	N	-
6	69	440077	-	-	-	-	N	-
6	82	187751	-	-	-	P	-	-
6	94	165143	-	-	-	P	-	-
6	99	76857	-	-	-	N	-	-
6	106	775062	-	-	-	P	-	-
6	121	1922084	-	-	-	P	-	-
6	123	280634	-	-	N	-	-	-
6	130	148254	-	N	-	-	-	N
6	132	634199	-	-	P	-	-	P
6	139	716540	-	-	-	-	P	-
6	141	1847488	-	-	-	-	N	P
6	145	2016368	-	-	P	-	-	-
6	145	2409985	-	-	N	-	-	-
6	146	455023	-	-	-	-	-	N
6	156	2519435	-	-	-	-	N	-
6	158	1961285	-	-	-	-	-	N
6	158	3474996	-	-	-	N	-	-
6	169	431267	-	-	P	-	-	-
6	176	1475957	-	-	-	-	-	N
6	176	1633242	-	-	-	P	-	-
6	209	25742	-	-	-	-	-	N
6	292	151856	-	N	-	-	-	-
6	514	1844	-	P	-	-	-	-
6	548	7882	P	-	-	-	-	-
6	705	6615	-	P	-	-	-	-
6	728	9316	N	-	-	-	-	-
6	729	1211	-	P	-	-	-	-
6	778	50766	-	-	-	P	-	N
6	1519	6549	-	-	-	-	P	P
NA	0	2	P	-	-	-	-	-
NA	0	11	N	-	-	-	-	-
NA	0	18	P	-	-	-	-	N
NA	0	18	-	-	N	P	-	-
NA	0	24	-	N	-	-	-	-
NA	0	33	-	-	-	P	-	-

NA	0	34	-	-	P	-	-	-
NA	0	37	-	-	N	-	-	-
NA	0	39	-	-	-	-	-	N
NA	0	47	-	-	-	-	N	-
NA	0	51	-	-	P	-	-	-
NA	0	52	-	-	-	P	-	-
NA	0	58	-	-	-	-	N	-
NA	0	86	-	-	-	-	-	N
NA	0	90	-	-	-	N	-	-
NA	0	91	-	-	-	-	-	P
