# Supplementary material for: Detecting Selection on Temporal and Spatial Scales: A Genomic Time-Series Assessment of Selective Responses to Devil Facial Tumor Disease
Source: PLoS One. 2016 Mar 1;11(3):e0147875. doi: 10.1371/journal.pone.0147875 (PMC4773136; doi:10.1371/journal.pone.0147875)
Supplement: S4 File — (PDF) [file pone.0147875.s004.pdf]

SI 4. Overview of SNPs under selection detected with demographic and time-series methods. SNPs under selection for each year (1999, 2004, 2009 and 2013) as identified with BAYESCAN and in individual populations assuming a) a small (N_e=50) and b) a large (N_e=500) effective population size identified with WFABC. For each SNP chromosome (Chr.), super-contig and position in super-contig are given. Direction of selection is given as positive (P) and negative (N). Only SNPs identified as being under selection with both methods are included.

	**Chr.**	**Super-contig**	**SNP position**	**1999**	**2004**	**2009**	**2013**	**Woolnorth**	**Arthur River**	**Narawntapu**	**Mt William**	**Freycinet**	**Forestier**
a)													
	2	67	673351	-	-	P	-	N	N	-	N	N	N
	5	43	1194068	-	-	P	-	-	-	P	-	-	-
b)													
	1	2	960133	-	P	P	-	-	-	-	P	-	-
	1	5	3478545	-	P	-	P	-	-	-	P	-	P
	1	12	876195	-	P	P	-	-	N	-	-	-	-
	1	34	2090729	-	-	P	-	-	-	-	N	-	-
	1	54	1481620	-	-	-	P	-	-	-	-	N	-
	1	124	537278	-	-	-	P	P	-	-	-	N	-
	1	259	73417	P	-	-	P	-	-	-	-	-	P
	1	259	2775110	P	P	P	P	-	-	-	-	P	-
	1	289	1692290	P	P	P	P	-	-	N	-	-	-
	1	297	1446547	-	-	-	P	-	-	N	-	-	-
	1	375	696764	-	-	P	P	-	N	-	-	-	N
	1	381	278795	-	-	-	P	-	-	-	P	-	-
	1	445	71025	-	P	P	P	-	-	-	-	-	N
	1	445	75680	-	P	P	P	-	-	-	-	-	N
	1	446	132042	-	-	-	P	-	-	-	N	-	-
	1	446	133260	-	-	-	P	-	-	-	N	-	-
	1	630	3069	P	P	P	P	-	N	-	-	-	-
	1	643	103647	-	P	P	P	-	-	-	N	-	-
	1	788	56371	-	-	-	P	-	-	-	-	-	P
	1	1246	33567	-	-	P	P	-	-	P	-	-	-
	1	1370	3906	-	-	P	P	-	-	-	-	P	-
	2	67	673351	-	-	P	-	-	P	N	P	-	N
	2	122	525086	-	-	P	-	-	-	N	-	-	-
	2	185	258819	-	-	P	-	-	-	P	-	-	-
	2	233	676222	-	-	P	-	-	-	P	-	-	-
	2	235	2941774	-	-	P	P	-	-	P	-	-	-
	2	254	173178	-	-	P	P	-	-	P	-	-	P
	2	256	209544	-	-	P	-	P	-	-	-	-	-
	2	279	1962190	P	-	-	-	-	-	N	-	-	-
	2	313	1149368	-	-	P	-	-	-	-	-	-	N
	2	451	570671	-	-	P	-	-	-	-	-	-	N
	3	93	249731	P	P	P	P	-	-	-	-	P	-
	3	109	1363279	-	P	-	-	-	-	P	-	-	-
	3	137	836212	-	-	P	-	P	-	-	-	-	-
	3	260	2537254	-	-	P	-	-	-	-	-	N	-
	3	310	1770399	-	-	P	P	-	-	-	-	-	N
	3	314	2784883	-	-	P	-	-	-	-	-	P	-
	3	357	217434	-	-	P	-	P	-	-	N	-	-

3	390	2536138	P	-	P	P	-	-	-	-	-	N
3	1483	4552	-	-	P	-	-	N	-	-	-	-
4	4	597847	-	-	P	-	-	-	-	N	-	-
4	15	1005633	-	-	P	P	-	-	-	-	-	N
4	15	1523425	-	-	P	P	-	-	-	P	-	-
4	17	3632032	-	P	P	-	-	-	-	P	-	-
4	60	1569690	-	-	-	P	-	-	-	-	-	P
4	77	2113411	-	P	P	P	P	-	-	-	-	-
4	103	1133697	-	P	P	-	-	-	-	N	-	-
4	127	1864609	P	-	-	-	-	-	-	-	P	-
4	155	685114	-	-	-	P	P	-	N	-	-	-
4	251	828774	-	P	-	-	-	-	-	-	N	-
4	298	71438	-	-	P	P	-	-	N	-	-	-
4	657	33346	-	-	P	P	-	-	-	N	-	-
4	657	127489	-	-	P	P	-	-	-	N	-	-
4	1826	196	-	P	-	P	-	-	-	P	-	-
5	43	1194068	-	-	P	-	-	-	-	P	-	-
5	50	1376047	-	-	-	P	-	-	-	-	P	-
5	63	1205307	-	-	-	P	-	-	-	-	-	N
5	464	46402	-	-	P	-	-	-	-	P	-	-
6	82	187751	-	-	P	P	-	-	-	P	-	-
6	94	165143	-	-	P	P	-	-	-	P	-	-
6	106	775062	-	-	-	P	-	-	-	P	-	-
6	145	2409985	-	P	-	P	-	-	N	-	-	-
6	156	2519435	-	-	P	P	-	-	-	-	N	-
6	728	9316	-	-	P	-	-	N	-	-	-	
