# Supplementary material for: Detecting Selection on Temporal and Spatial Scales: A Genomic Time-Series Assessment of Selective Responses to Devil Facial Tumor Disease
Source: PLoS One. 2016 Mar 1;11(3):e0147875. doi: 10.1371/journal.pone.0147875 (PMC4773136; doi:10.1371/journal.pone.0147875)
Supplement: S5 File — (PDF) [file pone.0147875.s005.pdf]

SI 5. SNPs under selection at each year detected with BAYESCAN assuming prior odds of 100. For each SNP chromosome (chr.), super-contig, SNP position in super-contig and direction of selection, which for all SNPs are positive (P).

Chr.	Super-contig	SNP position	1999	2004	2009	2013
1	5	3478545	-	P	-	P
1	24	2439039	-	-	-	P
1	30	456063	-	-	-	P
1	103	63790	-	-	P	-
1	239	2175315	-	-	P	-
1	259	53959	-	-	-	P
1	259	73417	P	-	-	P
1	259	2775110	-	-	-	P
1	281	786550	P	-	-	P
1	281	1194959	P	-	-	P
1	289	1692290	-	-	P	P
1	297	1446547	-	-	-	P
1	375	696764	-	-	P	-
1	381	278795	-	-	-	P
1	386	537974	-	-	-	P
1	445	71025	-	-	P	P
1	445	75680	-	-	P	P
1	630	3069	-	-	P	P
1	643	103647	-	-	-	P
1	788	56371	-	-	-	P
1	1246	33567	-	-	P	P
2	13	8347	-	-	P	P
2	67	673351	-	-	P	-
2	231	725912	-	-	-	P
2	233	676222	-	-	P	-
2	235	2941774	-	-	P	P
2	241	412933	-	-	-	P
2	253	2410314	-	-	-	P
2	254	173178	-	-	P	P
2	382	421209	-	-	P	P
2	449	170227	-	-	-	P
2	569	60916	-	-	P	P
2	658	62646	-	-	-	P
2	1150	21386	-	-	-	P
3	93	249731	-	-	P	P
3	94	718106	-	-	P	P
3	94	779170	-	-	P	P
3	109	1314065	-	P	P	-
3	109	1363279	-	P	-	-
3	310	1770399	-	-	P	P

3	387	2836116	-	-	P	-
3	388	3437445	-	-	P	-
3	390	2536138	-	-	P	P
3	760	37586	-	-	P	P
3	3680	908	-	-	P	-
4	15	1005633	-	-	P	P
4	17	3632032	-	-	P	-
4	23	331080	-	-	-	P
4	32	54662	-	-	P	P
4	60	1569690	-	-	-	P
4	61	345278	-	-	-	P
4	77	2113411	-	P	P	P
4	87	1670726	-	-	P	-
4	103	1133697	-	P	-	-
4	155	685114	-	-	-	P
4	181	101628	-	P	-	-
4	237	956702	-	-	P	-
4	276	1861899	-	-	-	P
4	281	706685	-	-	P	-
4	302	548044	-	-	P	P
4	657	33346	-	-	-	P
4	657	127489	-	-	P	P
4	1078	47296	-	-	-	P
5	10	76100	-	-	P	P
5	55	1312293	-	-	-	P
5	68	250972	-	P	P	P
5	82	124066	-	-	P	P
5	157	1444854	-	-	P	P
5	175	242072	-	-	-	P
5	182	227713	-	P	-	P
5	368	180120	-	-	P	-
5	374	40069	-	-	-	P
5	447	7205	-	-	P	P
5	523	19999	-	-	-	P
5	625	3774	-	-	P	P
6	83	554595	-	P	-	-
6	94	165143	-	-	-	P
6	121	808041	-	-	P	-
6	145	2409985	-	P	-	P
6	156	2519435	-	-	P	-
6	167	3269766	-	-	-	P
6	168	390402	-	-	P	-
NA	0	2	-	-	-	P
NA	0	18	-	P	P	P
NA	0	47	-	P	-	-
NA	0	91	-	-	-	P
